# Simplistic Software for Analyzing Mass Spectra and a Mixed Experimental‐Theoretical Database for Identifying Poisonous and Explosive Substances

**DOI:** 10.1002/jcc.70148

**Published:** 2025-06-25

**Authors:** Denis S. Tikhonov, Mikhail A. Kalinin, Alexander A. Maryewski, Aleksandr A. Avdoshin, Olgert Dallakyan, Nikita A. Vasilev, Egor A. Eliseev, Mandy Koch, Vladimir V. Rybkin, Denis G. Artiukhin

**Affiliations:** ^1^ Deutsches Elektronen‐Synchrotron DESY Hamburg Germany; ^2^ Free Moscow University Moscow Russia; ^3^ Institute of Chemistry Martin Luther University Halle‐Wittenberg Halle (Saale) Germany; ^4^ Institute of Physical Chemistry (IPC) Karlsruhe Institute of Technology Karlsruhe Germany; ^5^ Independent researcher; ^6^ Institute of Chemical Physics NAS RA Yerevan Armenia; ^7^ HQS Quantum Simulations GmbH Karlsruhe Germany; ^8^ Institut für Chemie und Biochemie Freie Universität Berlin Berlin Germany

**Keywords:** database, mass spectra, metric, molecular dynamics, substance identification

## Abstract

A recent increase in targeted attacks using chemical warfare agents by dictators and authoritarian regimes against politicians, journalists, and other civilians is a major concern. To aid the civil investigators in identifying poisonous substances in such cases, we developed an algorithm and a lightweight and simple‐to‐use software, ToxicMassSceptic, with a database of 400 electron ionization mass spectra entries, which include many poisonous and explosive agents. The identification relies on a window‐based reduction of the experimental spectra and four statistical metrics that are combined into a single metametric. The software also features automatic spectral background removal. Furthermore, we provide the workflow for increasing the size of this database by performing theoretical calculations of mass spectra with a molecular dynamics‐based approach. The accuracy of both the theoretical prediction workflow and ToxicMassSceptic is validated on the experimental spectra. Our results demonstrate that the proposed software package can aid in the preliminary identification of traces of poisonous and explosive substances.

## Graphical abstract

We present a new lightweight approach for analyzing mass spectra and a database oriented toward toxic and explosive compounds. The database is Ñ•ompliled from experimental and theoretically predicted electron‐ionization mass spectra. Our results demonstrate that the proposed software Ñ•an assist in the preliminary identification of poisonous and explosive substances.

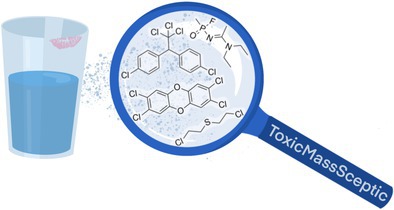

**Abbreviations**
AIST: National Institute of Advanced Industrial Science and Technology
EI: electron ionization
GC: gas chromatography
HPLC: high‐pressure liquid chromatography
IC: internal conversion
IEE: internal excess energy
KE: kinetic energy
KER: kinetic energy release
MD: molecular dynamics
MR: mean rank
MRR: mean reciprocal rank
MS: mass‐spectrometry
NIST: National Institute of Standards and Technology
NMR: nuclear magnetic resonance
PAHs: polycylcic aromatic hydrocarbons
XUV: extreme ultraviolet

## 1 Introduction

The Chemical Weapons Convention [1], which entered into force in 1997, marked a breakthrough in a long‐standing effort to end the production, storage, and eventual deployment of poisoning agents in a military setting. Despite its nearly universal adoption, multiple large‐scale assaults involving chemical weapons have occurred in the decades after the adoption, most notably in Syria (before [2] and after [3] its accession to the convention) and Iraq [4]. In a concerning development, nerve combat agents, originally designed for indiscriminate large‐area use, have been employed in attempts on the lives of individuals in urban environments. The most well‐known case is the Tokyo subway sarin attack, performed in 1995 by the Aum Shinrikyo cult, that killed 13 and injured more than 6000 people [5, 6]. In recent years, authoritarian regimes in Russia and North Korea [7, 8] have made targeted attempts at using various poisons to assassinate dissidents and critics [9, 10]. Thus, Russian democratic opposition leader Alexei Navalny [11, 12] and former Russian spy and double agent for British intelligence Sergei Skripal [13] were notoriously poisoned with the Novichok nerve agent, Ukrainian president Viktor Yushchenko was poisoned during his presidential campaign of 2004 by the TCDD agent [14], and an exiled relative of North Korea's supreme leader Kim Jong Un, Kim Jong‐nam [15], was killed using the VX nerve agent. Months after the attempt on Skripal, an unrelated British couple was poisoned with Novichok [16], apparently as collateral from a Russian attack.

Although in the aforementioned high‐profile cases the specific nerve agents were reliably identified, investigations into other apparent poisonings did not produce conclusive results on the nature of the chemical agents used. In cases of Russian regime critics Pyotr Verzilov [17], Dmitry Bykov [18], Vladimir Kara–Murza [19], the latter being poisoned on two separate occasions, or in a recent chain of poisonings of dissident Russian journalists and activists after the outbreak of Russian aggression against Ukraine [20], the used substances were not definitively established, which might be due to delays in samples collection and their analysis.

A range of methods exists to identify the presence of chemical warfare agents in the laboratory or the field. The most sensitive and informative of these are non‐portable techniques: Mass spectrometry (MS), nuclear magnetic resonance (NMR), and chromatographic methods, such as gas chromatography (GC) or high‐pressure liquid chromatography (HPLC), coupled to MS [21, 22, 23, 24, 25]. In their review on the detection and destruction of chemical warfare agents, Kim et al. [26] provide numerous examples of MS techniques being used to identify organophosphorus nerve agents and other toxins at very low concentrations, in some cases in vivo. In most MS techniques, the molecules present in the sample undergo fragmentation upon ionization, which makes interpretation of mass spectra a cumbersome task even when dealing with a clean individual substance, increasing the likelihood of failure to identify a compound in the probe. In real‐world forensic samples, often heavily contaminated and containing only traces of compounds, reliable identification becomes an exceedingly difficult task. Thus, a method to automatically identify poisons or other dangerous chemical compounds in mass spectra of impure samples is of great interest to a broad community of forensic experts, medical professionals, as well as independent sleuths. Since investigations are often conducted by individuals and teams with no technical education and at their own risk, we also note that a software piece to implement this method must be easy to install and operate without MS specialist knowledge.

Focusing on MS as the prime method to identify various species in experimental mixtures, we find ourselves with a wide selection of program tools for analyzing mass spectra. First of all, many producers of MS equipment provide accompanying software to be used with it. The MassHunter code by Agilent [27] is one such example. Secondly, the analysis software developed by the National Institute of Standards and Technology (NIST), such as the AMDIS (Automated Mass Spectrometry Deconvolution and Identification System) and MS Search [28, 29, 30, 31, 32] are commonly used. The drawback of these programs is that they are proprietary. As an alternative, there are also open‐source software, such as the ProteoWizard [33], matchms [34, 35], OpenMS/pyOpenMS [36, 37], and FastEI [38]. However, most of these packages require both advanced user experience and proficiency in MS. Therefore, these software packages can be hard to use for non‐experts.

Finding the reference spectra in the existing literature might also present a challenging problem. In the publicly accessible databases, such as those by The NIST Chemistry WebBook [39] or National Institute of Advanced Industrial Science and Technology (AIST) [40], experimental data for many substances are not present, for instance for the compounds described in the book by Mirzayanov [41]. There are some attempts to combine personal libraries of spectra, for example, FederEI [42], a federated library matching framework for EI‐MS. Another possible solution to this problem is to predict spectra from theory. Nowadays, various methods for such prediction exist. Among those are the machine‐learning‐based prediction algorithms, such as competitive fragmentation modeling (CFM) [43, 44, 45], rapid approximate subset‐based spectra prediction (RASSP) [46], and neural electron‐ionization mass spectrometry (NEIMS) [47]. In recent years, an algorithm to compute mass spectra by means of molecular dynamics (MD) simulations was proposed by Grimme [48]. This algorithm was used to predict the MS spectra, among others, of Tabun [49] and Novichok [50], experimental work therewith being greatly hindered by the inherent danger.

To address the outlined difficulties, we present a simple‐to‐use software package, ToxicMassSceptic, for the analysis of mass spectra, together with a database compiled from both MS experiments and theoretical computations, as well as the workflow for producing the theoretical mass spectra. We do not aim to outperform existing identification methods and libraries but rather to provide a simple and robust tool for preliminary substance identification that can aid low‐budget analytical laboratories and civil investigators. The article has the following structure. First, in Section 2, we introduce the methodology: The structure and sources of the database, the digital formats of the data, and algorithms and workflows to compute and assign mass spectra, including the spectral similarity metrics. Secondly, we discuss the theoretical computation of mass spectra and demonstrate applications of the methodology in Section 3. Finally, conclusions are outlined in Section 4.

## 2 Methods

### 2.1 Mass‐Spectroscopic Database

#### 2.1.1 Database Structure and File Formats

**FIGURE 1:**
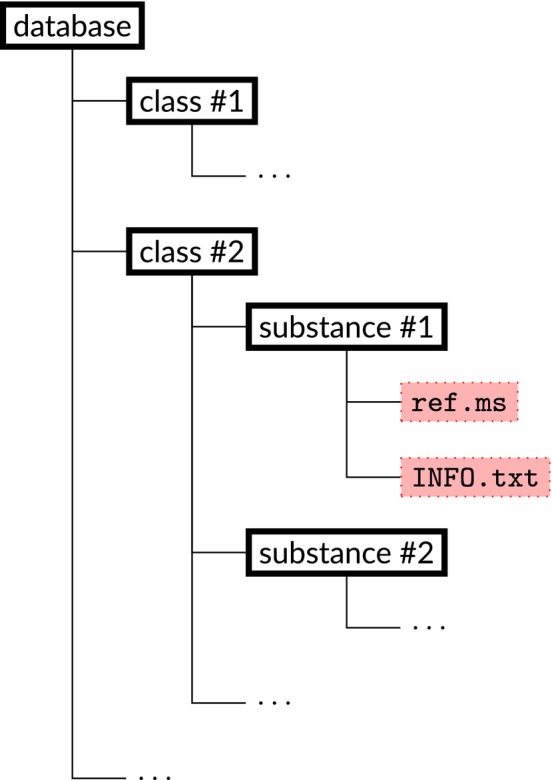
Schematic structure of the database with reference MS. The symbol “…” denotes similarly repeated structure.

Our database has to be easy to extend even by inexperienced users. Therefore, we store it as a set of nested directories with the structure shown in Figure 1. The top‐level directory (“database”) contains the subdirectories that name the class of substances (“class #1”, “class #2”, etc.). Each of the subdirectories (“substance #1”, “substance #2”, etc.) contains folders with data on the specific substance. The recommended naming of these folders is “[Brutto chemical formula in the Hill notation]_[common name of the substance].” For every substance, the “ref.ms” file is required, which contains the reference mass spectrum of the given compound. It is optional but strongly suggested to supplement an entry with a file “INFO.txt” that contains information about the substance, for example, common names, molar mass, links to substance Wikipedia and/or PubChem webpage, etc.

The classes of substances in the presented database and the number of entries in each are shown in Table 1. While the classification of substances is almost always self‐explanatory, assuming their separation into different chemical weapon agent types (blister agents, blood agents, chocking agents, lachrymators, and nerve agents) [51], environmental pollutants (such as polycyclic aromatic hydrocarbons (PAHs) [52, 53], per‐ and polyfluoroalkyl substances (PFAS), polychlorinated biphenyls (PCBs), and dioxines [54, 55]), a separate category (miscellaneous) had to be made to store different substances that did not fit into this arguably rigid framework.

**TABLE 1:** Classes of substances present in the database and the number of substances in each category ($N_{\text{sub}}$).

Class of substances	$N_{\text{sub}}$
AcidContaminants	9
Bisphenols	3
BlisterAgents	15
BloodAgents	6
Chlorophenols	7
ChokingAgents	9
Dioxines	15
Explosives	59
Herbicides	7
Lachrymators	5
Miscellaneous	169
NerveAgents	43
PAHs	16
PCBs	2
Pesticydes	31
PFASs	2
Phthalates	2

*Note:* In total, the database contains 400 entries, a few of which represent the same substances but different spectra.

The reference spectra of the molecules in the database (files “ref.ms”) are formatted as two‐column text files with pairs of numbers (x,y) in rows, where x is the integer mass‐over‐charge (m/z) position of the ion and y is the normalized intensity of the given ionic fragment in the MS; this format is usually denoted with an .xy file extension. The spectra in the “ref.ms” files have different normalization and are to be treated as not normalized, while normalization happens during runtime. For a molecule with a spectrum of N fragment ions $\left\{(x_{1},y_{1}),(x_{2},y_{2}),\text{…},(x_{N},y_{N})\right\}$, the intensities are normalized such that

(1) $$\overset{N}{\underset{i=1}{\sum }}y_{i}=100\%$$

#### 2.1.2 Sources of Experimental Mass Spectra

Our database of molecular species borrowed mainly from the following sources: The NIST Chemistry WebBook [39], Spectral Database for Organic Compounds SDBS [40] organized by the AIST, Japan, and University of Rhode Island Explosives Database [56]. Since the Chemistry WebBook removed the option to download numerical MS data, most of the information from this database was extracted by manually digitizing the graphs (for details of this procedure, see ESI). The spectra for the two Novichok species, A‐230 and A‐232, were digitized from [57] using WebPlotDigitizer software [58].

#### 2.1.3 Sources of Theoretical Mass Spectra

**FIGURE 2:**
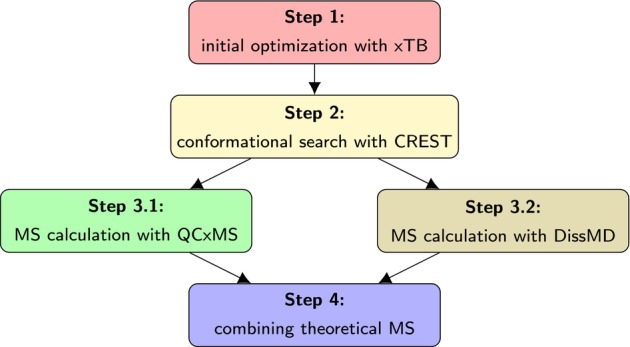
A general workflow scheme applied for the theoretical MS prediction for a given molecule.

Theoretical mass spectra were computed using the workflow shown in Figure 2. All quantum chemical calculations, including conformational search and the MS calculation, were done with the GFN2‐xTB method [59] as implemented in the xTB software [60], version 6.6.1. First, the initial molecular structure, obtained either from the NIST Chemistry WebBook, PubChem, or drawn in Jmol [61], was optimized with the xTB software. Then, conformational search was performed for this structure using CREST (version 2.12) [62, 63], except for conformationally‐rigid molecules. Subsequently, two augmented Born–Oppenheimer molecular dynamics (aBOMD) program packages were applied to calculate the theoretical mass spectrum of the lowest energy conformer: QCxMS (version 5.2.1) [48, 64, 65], an original approach by S. Grimme, and DissMD, a software [66, 67, 68] based on the same idea. A detailed comparison of those approaches can be found in Section 3. Finally, the spectra obtained by the two theoretical approaches described above were combined as arithmetic means.

In QCxMS, the default settings were applied. The molecules were ionized by electron ionization (EI) with kinetic energy of electrons equal to 70 eV. The spectra were then collected by PlotMS (version 6.1.0). Since DissMD only simulates laser ionization, the ionization of molecules was modeled with an extreme ultraviolet (XUV) photon of 70 eV energy. In both QCxMS and DissMD calculations, the GFN2‐xTB method was used to provide the potential energy surfaces for the aBOMD simulations, as this method was shown to be sufficiently accurate and computationally feasible for the mass spectra prediction [64, 69].

### 2.2 Mass‐Spectra Assigning Algorithm

#### 2.2.1 Window‐Function Based Assignment

The assignment was based on the assumption that there might be more than one species in the MS, which can be the case if the mixtures were not properly separated by chromatography or an alternative technique applied before the MS analysis. Therefore, the procedure involves finding only the relevant peaks in the tested spectrum to be compared with the reference database. For this, the window‐based metrics were employed as described in more detail in the following.

Let us assume that we are interested in the possibility of species A with known reference spectrum of N(A) peaks $\left\{(x_{1},y_{1}),(x_{2},y_{2}),\text{…},(x_{N(A)},y_{N(A)})\right\}$ to be present in the mixture. Intensities $y_{i}$ can be represented as an N(A)‐dimensional vector $y(A)=(y_{1},y_{2},\text{…},y_{N(A)})$. Note that we require all intensities to be positive ($y_{i}>0$ for i=1,…,N(A)) and normalized to 100% as seen from Equation (1). To make the comparison, we need to reduce the experimental dataset to an analogous N(A)‐dimensional vector of experimental intensities $\tilde{y}(B|A)=({\tilde{y}}_{1},{\tilde{y}}_{2},\text{…},{\tilde{y}}_{N(A)})$, where ${\tilde{y}}_{i}$ is the spectral intensity around $x_{i}=m_{i}/z_{i}$ in experimentally‐measured MS I(x) of unknown species or mixture B. To that end, we integrate the raw experimental MS I(x) with a window function $w(x|x_{i})$ for a given position $x_{i}=m_{i}/z_{i}$ and obtain non‐normalized intensities $\left(Y_{1},Y_{2},\text{…},Y_{N(A)}\right)$ as

(2) $$Y_{i}=\int_{0}^{+\infty }I(x)\cdot w(x|x_{i})dx$$

where $w(x|x_{i})$ is nonzero only in the vicinity of $x_{i}$. This mathematical operation essentially sums up the spectral intensity near an expected position $x_{i}$ into a single value. Applying this transformation to every peak i in the reference spectrum I(x) and subsequently normalizing resulting values $Y_{i}$ such that

(3) $${\tilde{y}}_{i}=100\%\times \frac{Y_{i}}{\sum_{j=1}^{N(A)}Y_{j}}$$

we obtain experimental intensities ${\tilde{y}}_{i}$ at the discretized positions $x_{i}=m_{i}/z_{i}$ of the reference dataset.

Alternatively, if the experimental MS is presented in the form of discrete peaks, the integration procedure is replaced by the summation, namely

(4) $$Y_{i}=\overset{M}{\underset{k=1}{\sum }}I_{k}\cdot w(x_{k}|x_{i})dx$$

where index k runs over all M peaks with intensities $I(x_{k})=I_{k}$ identified in the experimental MS by the spectrometer's software.

In our program code, we implemented two types of window functions $w(x|x_{i})$: A rectangular window,

(5) $$w(x|x_{i})=\left\{\begin{array}{cc} 1,\; & |x-x_{i}|\leq \sigma /2\\ 0,\; & |x-x_{i}|>\sigma /2 \end{array}\right.$$

and Gaussian window

(6) $$w(x|x_{i})=\exp\left(-\frac{{\left(x-x_{i}\right)}^{2}}{2\sigma^{2}}\right)$$

where σ is the width of the given window in m/z units. By default, the Gaussian window with σ=1/2 is employed.

#### 2.2.2 Assignment Metric

After defining the window‐based reduction scheme of experimental data, we can discuss the route to identifying chemical species in our spectrum. To that end, we rely on a metametric, which is composed of several deterministic metrics. Thus, the simplest metric N(B|A) that can be defined for a given reference spectrum A is the number of lines present in both A and B. It reads

(7) $$N(B|A)=\overset{N(A)}{\underset{i=1}{\sum }}\theta ({\tilde{y}}_{i}-c)$$

where c>0 is a small threshold (in our case, $c=10^{-15}$) for numerical comparison of real numbers and θ(x) is the Heaviside step function of the form

(8) $$\theta (x)=\left\{\begin{array}{cc} 1,\; & x>0\\ 0,\; & x\leq 0\; \end{array}\right.$$

The expression in Equation (7) can be normalized by the total number of lines in the reference spectrum N(A) to produce the relative number of lines, that is,

(9) $$P(B|A)=\frac{N(B|A)}{N(A)}$$

More sophisticated metrics should also account for the distribution of fragment intensities. For this purpose, two sets of normalized values y(A) and $\tilde{y}(B|A)$ can be treated as probability distributions. Thus, standard statistical distances for probability distributions can be employed. We chose four such measures: Kullback–Leibler divergence ($D_{\text{KL}}$) [70], Bhattacharyya distance ($D_{\text{B}}$) [71], Hellinger distance ($D_{\text{H}}$) [72], and cosine distance ($D_{\text{C}}$). In our case of two spectra, A and B, these four measures are given as [73, 74]

(10) $$D_{\text{KL}}(B|A)=\overset{N(A)}{\underset{i=1}{\sum }}{\tilde{y}}_{i}\cdot \ln\left(\frac{{\tilde{y}}_{i}}{y_{i}}\right)$$

(11) $$D_{\text{B}}(B|A)=-\ln\left(BC(B|A)\right)$$

(12) $$D_{\text{H}}(B|A)=\sqrt{1-BC(B|A)}$$

(13) $$D_{\text{C}}(B|A)=1-\frac{\sum_{i=1}^{N(A)}y_{i}{\tilde{y}}_{i}}{\sqrt{\left(\sum_{i=1}^{N(A)}{\tilde{y}}_{i}^{2}\right)\cdot \left(\sum_{i=1}^{N(A)}y_{i}^{2}\right)}}$$

respectively. In Equations (11) and (12), BC is the so‐called Bhattacharyya dimensionless coefficient [71, 75] given by

(14) $$BC(B|A)=\frac{1}{100\%}\overset{N(A)}{\underset{i=1}{\sum }}\sqrt{{\tilde{y}}_{i}\cdot y_{i}}$$

Here, the division by 100% is motivated by the fact that BC is defined for probability distributions normalized to 1. The three chosen measures of similarities for probability distributions from Equations ((9), (10), (11), (12)) require that components of the vector $\tilde{y}(B|A)$ are non‐negative. Note that Equations ((10), (11), (12)) are undefined for N(B|A)=0, which corresponds to the case of the species not being present in the spectrum.

The combined metametric is then constructed from Equations ((9), (10), (11), (12), (13)) such that

(15) $$\begin{array}{c} \begin{array}{lll} D_{\text{meta}}(B|A) & = & \frac{1}{P(B|A)\times \overset{N(A)}{\underset{j=1}{\sum }}Y_{j}}\\ & & \times \left(\frac{D_{\text{KL}}(B|A)}{\varsigma_{\text{KL}}}+\frac{D_{\text{B}}(B|A)}{\varsigma_{\text{B}}}+\frac{D_{\text{H}}(B|A)}{\varsigma_{\text{H}}}+\frac{D_{\text{C}}(B|A)}{\varsigma_{\text{C}}}\right)\; \end{array} \end{array}$$

where $Y_{j}$ is the non‐normalized experimental intensity given by Equations (2) or (4) and $\varsigma_{X}$ is the standard deviation of the given metric X= KL, B, H, and C, computed over the whole available dataset as

(16) $$\begin{array}{c} \begin{array}{l} \varsigma_{X}=\sqrt{\left\langle D_{X}^{2}\right\rangle -{\left\langle D_{X}\right\rangle }^{2}}\\ \;=\sqrt{\frac{1}{N_{\text{d}}}\sum_{A}D_{X}^{2}(B|A)-{\left(\frac{1}{N_{\text{d}}}\sum_{A}D_{X}^{2}(B|A)\right)}^{2}} \end{array} \end{array}$$

where index A runs over all spectra in the database and $N_{\text{d}}$ is the number of such spectra. The value of $D_{\text{meta}}(B|A)$ from Equation (15) tends to zero if the two spectra A and B are similar and increases with the growing dissimilarity of the experimental spectrum from the reference. Although Bhattacharyya and Hellinger distances provide the same relative ranking of substances, it can be advantageous to use both in the metametric, as they might have different sensitivity at different values of the Bhattacharyya dimensionless coefficient BC.

#### 2.2.3 Background Removal Algorithm

Experimentally measured spectra can contain signals from the background. This may result in empty areas of a spectrum producing negative intensities when using Equations (2) and (4). To avoid that, basic filtering of the experimental MS signal I(x) can be performed. The simplest and most robust approach is probably a visual determination of the noise threshold level $I_{\text{thr}}$, and setting all the values $I(x)\leq I_{\text{thr}}$ to zero. However, a crude automatic routine can also be designed (e.g., see [76]) assuming that non‐zero peaks occupy only a minor part of the spectrum in all available m/z ranges and that the baseline signal is I=0. To that end, we represent a spectrum in a discretized form with lines $I_{1},I_{2},\text{…},I_{M}$. Then, the following procedure can be employed.

1. Calculate the standard deviation of I(x) from baseline (I=0) as ${\text{SD}}_{0}=\sqrt{\frac{1}{M}\sum_{k=1}^{M}I_{k}^{2}}$.
2. Consider only values $I_{k}<q\cdot {\text{SD}}_{0}$, with q≥1 being an arbitrary selectivity coefficient, forming a new set $I_{1}^{\left(1\right)},I_{2}^{\left(1\right)},\text{…},I_{M_{1}}^{\left(1\right)}$, where the upper index “(1)” indicates the iteration number and $M_{1}\leq M$ is the number of elements in the new set.
3. Calculate the new standard deviation as ${\text{SD}}_{1}=\sqrt{\frac{1}{M_{1}}\sum_{k=1}^{M_{1}}{\left(I_{k}^{\left(1\right)}\right)}^{2}}$.
4. Repeat steps 2 and 3 until the number of elements in the set remains constant or a maximum number of iterations p is reached.
5. Set values of the original mass spectrum below the final threshold $q\cdot {\text{SD}}_{p}$ to zero.

This automatic background removal procedure is implemented in our program code, with the default number of steps p=3 and selectivity coefficient q=1.5.

### 2.3 Software

The program code called ToxicMassSceptic is written in Python version 3.8 for the Linux, MacOS, and MS Windows operational systems, distributed under an open source Apache License version 2.0 [77], and is managed using the version control system GIT [78] by the provider GitLab [79]. The source code is available in the Gitlab repository [80]. The list of program requirements includes Python packages such as numpy [81] and matplotlib [82]. The code has a clear version number and is accompanied by two types of documentation: (i) a README file in the Markdown format outlining external dependencies, package structure as well as the installation procedure and (ii) an automatically generated Doxygen [83] code documentation describing all constituting objects and functions. The package‐management system PIP3 [84] governs the installation procedure. The code is aimed to be fully unit‐tested. To that end, the package unittest [85] is employed. The current code design enables the use of our program as an external Python library as well as through a command‐line interface.

**FIGURE 3:**
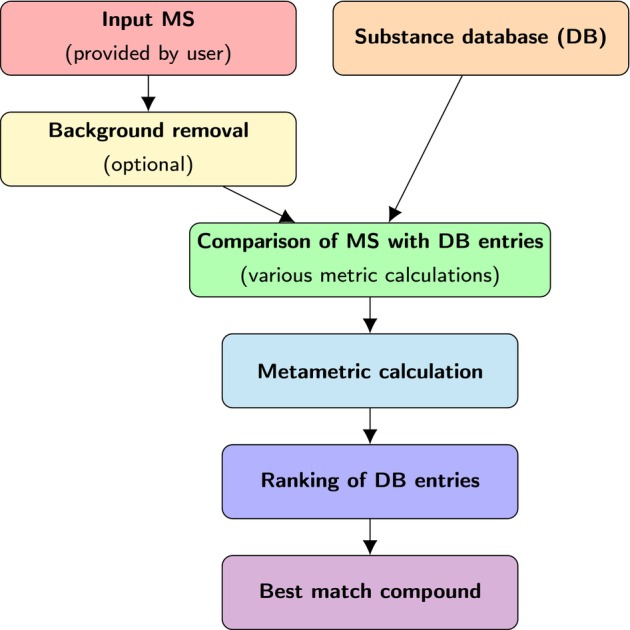
Flowchart of the ToxicMassSceptic software workflow. Details are given in the text.

The flowchart of the ToxicMassSceptic work and usage is given in Figure 3. First, the user needs to provide a spectrum, which can then be passed, by request, through the background removal procedure described in Section 2.2.3. Then, the database is loaded, and the comparison of the unknown spectrum with the database entry begins. During this step, the four metrics described in Section 2.2.2 are computed for each substance. After all the metrics are known, the metametric from Equation (15) for each database substance is computed, as it requires a spread of each metric throughout every database entry as seen from Equation (16). Finally, the database entries are sorted by the metametric value, and the best matching substance is given.

### 2.4 Statistical Analysis of Results

Let us assume that the user is interested in testing $N_{\text{trials}}$ number of different mixtures B. Each such ith mixture $B_{i}$ contains a compound $A_{\text{true}}^{i}$, which is also present in the database. Furthermore, we assume that for each sample $B_{i}$, the top‐K matching candidates $A^{i}=\{A_{1}^{i},A_{2}^{i},\text{…},A_{K}^{i}\}$ are suggested by our algorithm based on the metrics introduced above in Section 2.2.2. Here, each set $A^{i}$ is sorted in descending order such that its first element is the most probable match. Therefore, index j denotes the rank of compound $A_{j}^{i}$, that is, $j=R(A_{j}^{i})$, with lower ranks being preferable. Then, the following scores can be introduced to assess the performance of our algorithm.

1. *Top‐* K *accuracy* (also known as *Hit rate at rank* K), which is equal to the number of trials with the correctly identified compound being present in top‐best K candidates $N_{\text{in top‐}K}$ divided by the total number of trials $N_{\text{trials}}$ and multiplied by 100%, that is,
(17) $$\text{top}\text{−}K\;\text{accuracy}=\frac{N_{\text{in top‐}K}}{N_{\text{trials}}}\times 100\%$$
2. *Mean reciprocal rank (MRR)*, defined as
(18) $$\text{MRR}=\frac{1}{N_{\text{trials}}}\overset{N_{\text{trials}}}{\underset{i=1}{\sum }}\frac{1}{R(A_{\text{true}}^{i})}\times 100\%$$
  where $R(A_{\text{true}}^{i})$ is the rank of the correctly identified compound $A_{\text{true}}^{i}$ in trial i.
3. *Mean rank (MR)*, defined as
(19) $$\text{MR}=\frac{1}{N_{\text{trials}}}\overset{N_{\text{trials}}}{\underset{i=1}{\sum }}R(A_{\text{true}}^{i})$$

The top‐K score from Equation (17) shows how often the correctly identified compound was present in the K most probable candidates predicted by the program code, whereas MRR from Equation (18) evaluates the ability of the code to assign low ranks to relevant chemical compounds. In the case of an ideal assignment, when correct compounds always occupy the very top of the suggestion list, both scores are equal to 100%. The MR score from Equation (19) is closely related to MRR, but is equal to or greater than 1.0 and tends toward 1.0 for better‐performing recommendation systems.

## 3 Results and Discussion

### 3.1 Mass‐Spectra Prediction Workflow

Predicted mass spectra presented in this work were computed using either QCxMS or DissMD. The latter is a part of the PyRAMD package [66, 86, 87]. Both algorithms employ Born–Oppenheimer molecular dynamics (BOMD), as proposed by S. Grimme in his seminal paper [48]. Before discussing our results, we first compare the two approaches.

**FIGURE 4:**
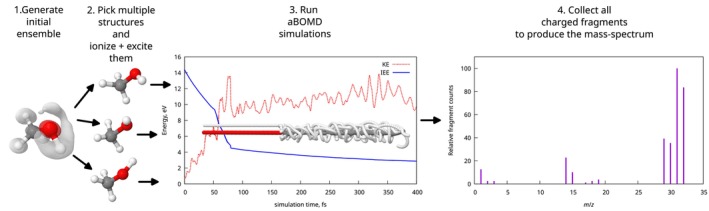
Graphical representation of a mass spectra simulation using aBOMD approach.

A graphical representation of an aBOMD‐based theoretical workflow for an MS spectrum prediction is depicted in Figure 4. First, multiple molecular geometries are generated, representing the gaseous ensemble of molecules in the spectrometer. Those structures are then used as initial points to start BOMD dynamics for ions. To include electronic excitation effects, the BOMD dynamics are perturbed (or augmented) by the kinetic energy influx from an external energy reservoir, producing an BOMD trajectory. This energy, referred to as the internal excess energy (IEE), and the ion charge are ascribed according to the ionization procedure. If, upon the aBOMD trajectory propagation, a dissociation of the molecule is detected, the parent ion trajectory is stopped, and new aBOMD trajectories for the products are initiated by sharing the charge and IEE of the parent ion between fragments. Then, these trajectories of the daughter ions are propagated further. Finally, the mass spectra are computed from the ensemble of MD trajectories by counting the final products.

Despite this scheme's general simplicity, a few crucial components in the algorithm define the simulation behavior. The QCxMS and DissMD use two completely different approaches to generate initial conditions. In the QCxMS, the thermostated MD of the neutral molecule is performed to sample the initial structures and their velocities. In the DissMD, the simplified Wigner sampling [87, 88] approach from a user‐provided geometry is used, which, in principle, can include some of the nuclear quantum effects [89] for the lighter nuclei such as hydrogens. Furthermore, these two approaches also differ greatly in the ionization procedure and the assignment of the IEE. In QCxMS, an arbitrary Poisson‐like distribution is employed [64, 90]

(20) $$P(\text{IEE})=\frac{\exp\left(c\cdot \text{IEE}\cdot (1+\ln\left(b/(c\cdot \text{IEE})\right)-b\right)}{a\cdot \text{IEE}+1}$$

where P(IEE) is the probability of the ion to have the value of IEE upon ionization, whereas a=0.2eV, b=1eV, and $c=1/N_{\text{ve}}$ are pre‐defined parameters with $N_{\text{ve}}$ being the number of valence electrons in the system. In the DissMD, however, an approach based on the electronic density of the states is used. Upon applying the maximum entropy principle and energy conservation to molecular ionization, one arrives at the following distribution [67]:

(21) $$P(\text{IEE})=\text{DoS}(\text{IEE})\cdot {\left(E_{\text{i}}-\text{IP}-\text{IEE}\right)}^{\frac{N_{\text{f}}}{2}-1}$$

where DoS(IEE) is the electronic density of states of the ion, $E_{\text{i}}$ is the total energy of the ionization event, IP is the sum of ionization potentials to reach a given ionization state, and $N_{\text{f}}$ is the number of degrees of freedom for the leaving particles. For the photoionization, which is the only available case in DissMD, $E_{\text{i}}=mh\nu$ and $N_{\text{f}}=3\cdot N_{\text{re}}$. In these expressions, m is the number of absorbed photons, $h=6.626\times 10^{-34}\;\text{J}\cdot \text{s}$ is the Planck constant, ν is the photons' frequency, and $N_{\text{re}}$ is the number of electrons removed upon ionization ($N_{\text{f}}=3$ for single ionization, $N_{\text{f}}=6$ for double ionization, etc.). Note, however, that Equation (21) can still be applied for the electron impact ionization. In this case, $E_{\text{i}}$ is the kinetic energy of the electrons and $N_{\text{f}}$ is set to $3\cdot (N_{\text{re}}+1)$ to account for the leaving ionizing particle's degree of freedom. Unlike in the first version of the software, in which the explicitly computed excited states were used to obtain the electronic density of states [86], the current version of the DissMD uses a simplified heuristic model based on the Van‐der‐Waals volume and surface to approximate $\text{DoS}(\text{IEE})\propto {\text{IEE}}^{n}$ as a power function with a single parameter n. In this case, Equation (21) reduces to a beta‐distribution [67].

The third crucial component of the simulation is the rate of internal conversion (IC), showing how fast the IEE decays into nuclear motions. For this purpose, the QCxMS uses the energy‐gap law in the form [64]

(22) $$k_{\text{IC}}^{-1}=\overset{M}{\underset{j>i}{\sum }}\frac{k_{\text{h}}}{N_{\text{ve}}}\exp\left(\alpha (\varepsilon_{i}-\varepsilon_{j})\right)$$

where $k_{\text{h}}=2\;\text{ps}$ and $\alpha =0.5\;{\text{eV}}^{-1}$ are constrants, $\varepsilon_{i}$ is the energy of an i‐th orbital, and M is the total number of orbitals. Contrary to that, in the DissMD, a classical model of hot electrons with kinetic energy of IEE colliding with motionless nuclei is employed. In the DissMD prototype, a similar algorithm, based on an idea of electron‐nuclear collision‐induced IC, was used to compute the IC rates using the atomic electronic densities through the plasma frequency estimated from atomic charges [67]. However, in the newer code, it was replaced with a simplified model for the rate of such collisions is given as [68]

(23) $$k_{\text{IC}}=\kappa \frac{\sqrt{m_{\text{e}}\text{IEE}}}{m_{\text{amu}}(L_{0}+L_{\text{mol}})}N_{\text{n}}N_{\text{e}}$$

where $N_{\text{e}}$ and $N_{\text{n}}$ are the total number of electrons and nuclei in the ion, respectively, $m_{\text{e}}$ is the electron mass, $m_{\text{amu}}$ is the atomic mass unit (dalton), $L_{\text{mol}}$ is the molecular length (atomic‐charge‐product‐weighted sum of all chemical bonds, determined from the covalent radii of atoms), $L_{0}=5\;Å$ is the regularizing parameter, and κ≈1.28 is the fitted parameter based on the available experimental data [68].

When the dissociation is detected, the QCxMS and DissMD again proceed in a different fashion. The DissMD follows a direct route: Upon the detection of dissociation of ion ${\text{M}}^{q+}$ into fragments A and B, it calculates the energies of several channels

(24) $${\text{M}}^{q+}\to {\text{A}}^{q_{\text{A}}+}+{\text{B}}^{q_{\text{B}}+}$$

that satisfy the charge conservation $q_{\text{A}}+q_{\text{B}}=q$. Upon dissociation, the channels with non‐negative kinetic energy release (KER) are assigned a probability proportional to this KER value. Subsequently, one of these channels is randomly chosen according to those probabilities. This leads to a speedup in the calculation, as the neutral fragments are not propagated. However, this approach requires a larger number of trajectories to be computed. In the QCxMS, a concept of statistical charge, or statistical weighing, is used. In this approach, the MD is carried out for all fragments, but their associated intensities depend on the weight, which is determined as [64]

(25) $$C_{i}=\frac{\exp\left(-\frac{{\text{IP}}_{j}}{k_{\text{B}}T}\right)}{\sum_{j}\exp\left(-\frac{{\text{IP}}_{j}}{k_{\text{B}}T}\right)}$$

with indices i and j running over the number of fragments, ${\text{IP}}_{j}$ being the ionization potential of a given fragment, $k_{\text{B}}$ being Boltzmann constant, and $T=\text{KE}/(3k_{\text{B}}N_{\text{n}})$ being the instant temperature of nuclei, as computed from their kinetic energy (KE). With these fragment weights, it is also possible to directly apply the isotopic distribution in the post‐analysis, while the DissMD requires running simulations with different isotopes.

**FIGURE 5:**
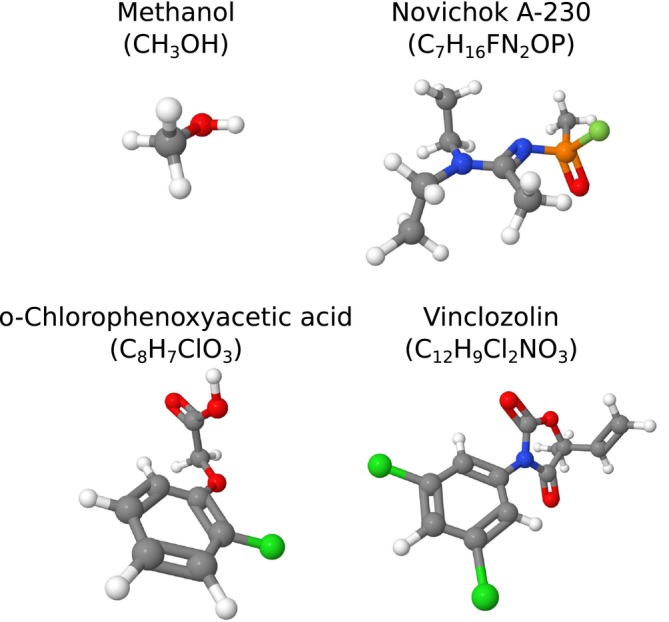
The most stable conformers of four test molecules used in the theoretical mass‐spectra prediction.

To demonstrate the predictive capabilities of the aBOMD‐based approach for computing the mass spectra, we took four molecules, for which we had the available spectra: Methanol ($CH_{3}\mathrm{OH}$), novichok A‐230 ($C_{7}H_{\mathrm{16}}FN_{2}\mathrm{OP}$), o‐chlorophenoxyacetic acid ($C_{8}H_{7}\mathrm{Cl}O_{3}$), and vinclozolin ($C_{\mathrm{12}}H_{9}Cl_{2}NO_{3}$). Structures of the most stable conformers of these molecules, according to CREST, can be found in Figure 5. As a metric to judge the similarity between spectra, we chose the number of peaks from the reference spectrum from Equation (7), the Kullback–Leibler divergence given in Equation (10), and the Bhattacharyya distance from Equation (11).

**FIGURE 6:**
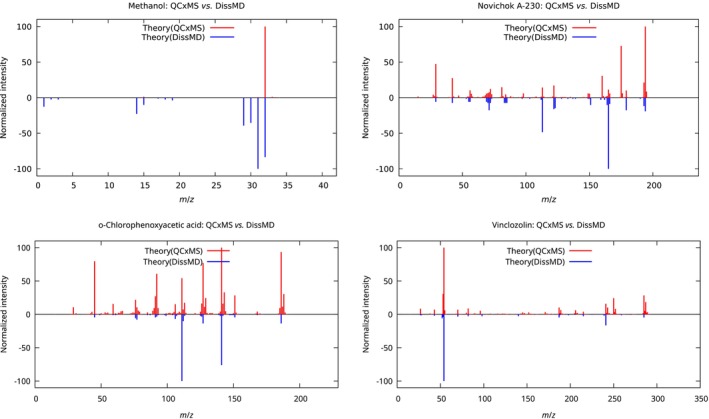
Comparison of the two theoretical mass spectra computed with QCxMS or DissMD software, for four test molecules (methanol, novichok A‐230, o‐chlorophenoxyacetic acid, and vinclozolin from Figure 5).

**FIGURE 7:**
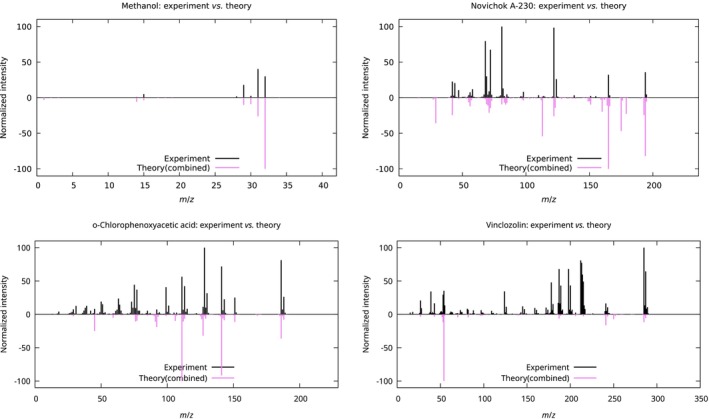
Comparison of the combined theoretical mass spectrum with the experimental one from the database for four test molecules (methanol, novichok A‐230, o‐chlorophenoxyacetic acid, and vinclozolin from Figure 5).

The results of our comparison are demonstrated in Figures 6 and 7, and in Table 2. It is clear that the QCxMS, as the software specifically designed for EIMS predictions, outperforms DissMD. Nevertheless, in three out of four cases, DissMD provided extra fragments that were missing in the QCxMS predictions. In all cases, the combination of both methods allowed us to cover more than 80% of lines from experimental spectra. However, the relative intensities of the peaks are not always perfect, which can be a result of wrong ionization conditions in the simulations. Nevertheless, we can confirm the conclusions from previous studies in [49, 50], stating that it is possible to use theoretically predicted mass spectra for the assignment of species with absent experimental reference spectra.

**TABLE 2:** Comparison of theoretically predicted mass spectra with their experimental reference counterparts from the database. The number of lines $N_{\text{lines}}$ is calculated via Equation (7), while $N_{\text{ref}}$ is the total number of peaks in the reference spectrum. The metrics $D_{\text{KL}}$ and $D_{\text{B}}$ are those given in Equations (10) and (11).

Spectrum	$N_{\text{lines}}/N_{\text{ref}}$	P, %	$D_{\text{KL}}$, %	$D_{\text{B}}$
Methanol (${\text{CH}}_{3}\text{OH}$)				
QCxMS	9/16	56.2	109.90	0.47
DissMD	10/16	62.5	29.02	0.06
Combined	13/16	81.2	36.98	0.10
Novichok A‐230 (${\text{C}}_{7}{\text{H}}_{\mathrm{16}}{\text{FN}}_{2}\text{OP}$)				
QCxMS	46/52	88.5	90.34	0.24
DissMD	17/52	32.7	180.01	0.60
Combined	46/52	88.5	117.12	0.32
o‐Chlorophenoxyacetic acid (${\text{C}}_{8}{\text{H}}_{7}{\text{ClO}}_{3}$)				
QCxMS	118/129	91.5	104.03	0.28
DissMD	30/129	23.3	137.05	0.56
Combined	120/129	93.0	98.48	0.30
Vinclozolin (${\text{C}}_{\mathrm{12}}{\text{H}}_{9}{\text{Cl}}_{2}{\text{NO}}_{3}$)				
QCxMS	84/105	80.0	122.95	0.42
DissMD	19/105	18.1	235.75	0.85
Combined	86/105	81.9	166.31	0.50

However, we would also claim that new software is probably due to development that would take the best algorithmic solutions from the QCxMS and DissMD. For the ionization stage, it makes more sense to assign the IEE from a physically sound model from Equation (21). For computing the IC rate, one might use a better model of the electron‐phonon coupling. One such possibility is demonstrated in [91, 92], where the rate is calculated based on the Fermi–Dirac distribution and orbital overlaps for the two consecutive MD steps. For the treatment of dissociation, the QCxMS approach appears more suitable. However, instead of using the heuristically defined weights from Equation (25), it would make more sense to use a modified version of the model introduced in [93], as it takes into account not only the ionization energies of fragments, but also the electron affinities, and the dissociation energies.

### 3.2 Performance Tests With Simulated Data

The ToxicMassSceptic features are subject to unit tests, ensuring the code works as expected. One of the production test trials the performance of the code in the presence of noise and additional substances. Here, we perform the testing based on the undecayed substances in our database, simulating mixtures taken directly from the environment, rather than from biological samples. This is due to the fact that the analysis of biological substances usually requires the use of liquid chromatography and searching for metabolites, which can be known only from in vitro studies (see, e.g., [24]). The biochemical degradation pathways of such compounds are highly unlikely to be found in publicly available literature in sufficient amounts to train empirical models. The theoretical prediction of those products from first principles is doubtful due to the sheer complexity of the problem. Therefore, without a proper database, we do not anticipate using ToxicMassSceptic directly for biological samples.

We model the species' spectra with Gaussian‐shaped peaks with randomly chosen standard deviation, that is, in the range between 0.05 and 0.1m/z. We take the mass spectrum of a randomly chosen species from the database and generate a spectrum in the m/z range from 0 to 500 with 2000 points. Then, we add a background that consists of two components. First, the signal of the substance is mixed with a spectrum composed of signals from benzene ($C_{6}H_{6}$), oxygen ($O_{2}$), nitrogen ($N_{2}$), carbon dioxide ($CO_{2}$), and farnesene ($C_{\mathrm{15}}H_{\mathrm{24}}$), one of sesquiterpenes. The relative amounts of the background species are randomly chosen between 0.1 and 0.2. Then, a random uniformly distributed noise is added on top of that with a signal‐to‐noise (S/N) level randomly chosen from the interval between S/N=100 and S/N=1000. Then, this generated spectrum is passed through our assignment algorithm, including the background removal and the rating of the actual compound, which is stored. The mean rating of the spectra upon multiple trials should not exceed an MR (Equation 19) threshold, which, in our case, is set to five. The current version of the software routinely passes this test.

To further demonstrate the performance of our code and compare different metrics, we carried out assignments of 500 randomly generated spectra. To that end, we modified the settings described above by lowering the allowed signal‐to‐noise level to 5≤S/N≤100, and additionally allowing peak intensities to vary by ±50% and their positions to be shifted by ±0.2m/z. The assignment was repeated 48 times, leading to 24,000 trials in total and allowing us to compute the mean values and standard deviations for statistical parameters from Equations ((17), (18), (19)). The results of this analysis are shown in Table 3. As can be seen, the worst top‐1 result is obtained using the cosine distance $D_{\text{C}}$, reaching an accuracy level of only about 30%. The performance of other metrics is much higher and varies from about 55% to 91%. Similar trends are observed for the MRR and MR scores. The use of the proposed metametric $D_{\text{meta}}$ was found to produce results of the highest quality in all cases.

**TABLE 3:** Performance of ToxicMassSceptic for simulated data. Results for the top‐K from Equation (17) and MRR from Equation (18) scores are given in %. The MR is given according to Equation (19).

	Top‐1	Top‐3	Top‐5	Top‐10	MRR	MR
$D_{\text{meta}}$	91±1	98.6±0.6	98.8±0.4	99.0±0.3	94.9±0.8	1.8±0.4
$D_{\text{KL}}$	55±2	87±2	94±1	96.9±0.9	72±2	3.3±0.7
$D_{\text{B}}$	61±2	92±1	97.0±0.9	98.7±0.7	77±1	2.1±0.3
$D_{\text{H}}$	61±2	92±1	97.0±0.9	98.7±0.6	77±1	2.1±0.3
$D_{\text{C}}$	30±2	69±2	84±2	95±1	53±2	4.1±0.5

### 3.3 Performance Test With Experimental Noisy Dataset

As an example of the mass spectra with noisy background, we took the strong‐field‐induced mass spectra of a tree‐ring PAH fluorene ($C_{\mathrm{13}}H_{\mathrm{10}}$), which are openly available from [94]. Since fluorene is in the database, and the laser‐induced fragmentation patterns look similar to those obtained with EI, we simply tested the identification of the species with the mass spectra obtained using different laser peak powers (from $1.5\times 10^{13}$ to $6.8\times 10^{13}\;\text{W}/{\text{cm}}^{2}$). In all of the cases, the automatic background removal was applied.

**FIGURE 8:**
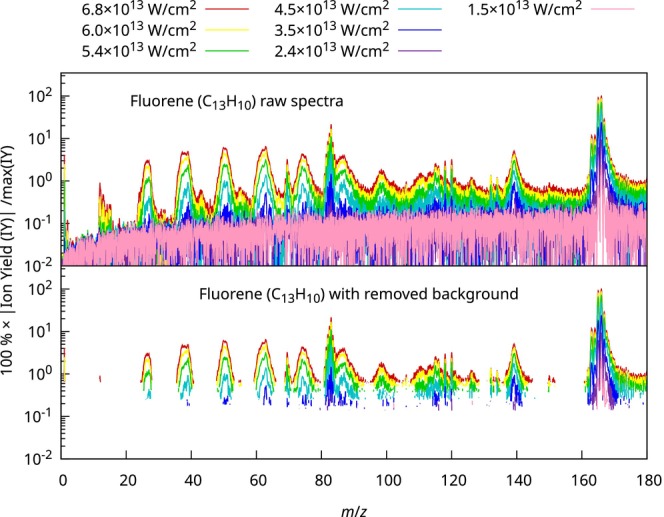
Experimental mass spectra of fluorene ($C_{\mathrm{13}}H_{\mathrm{10}}$) obtained by strong‐field ionization with ultrashort laser pulses of varied peak intensity. The top figure shows raw experimental spectra, while the bottom one is after background removal. Note that the logarithmic scale on the absolute intensity is used for the y‐axis, and the curve disappearance in the bottom figure means that the signal is zero.

**FIGURE 9:**
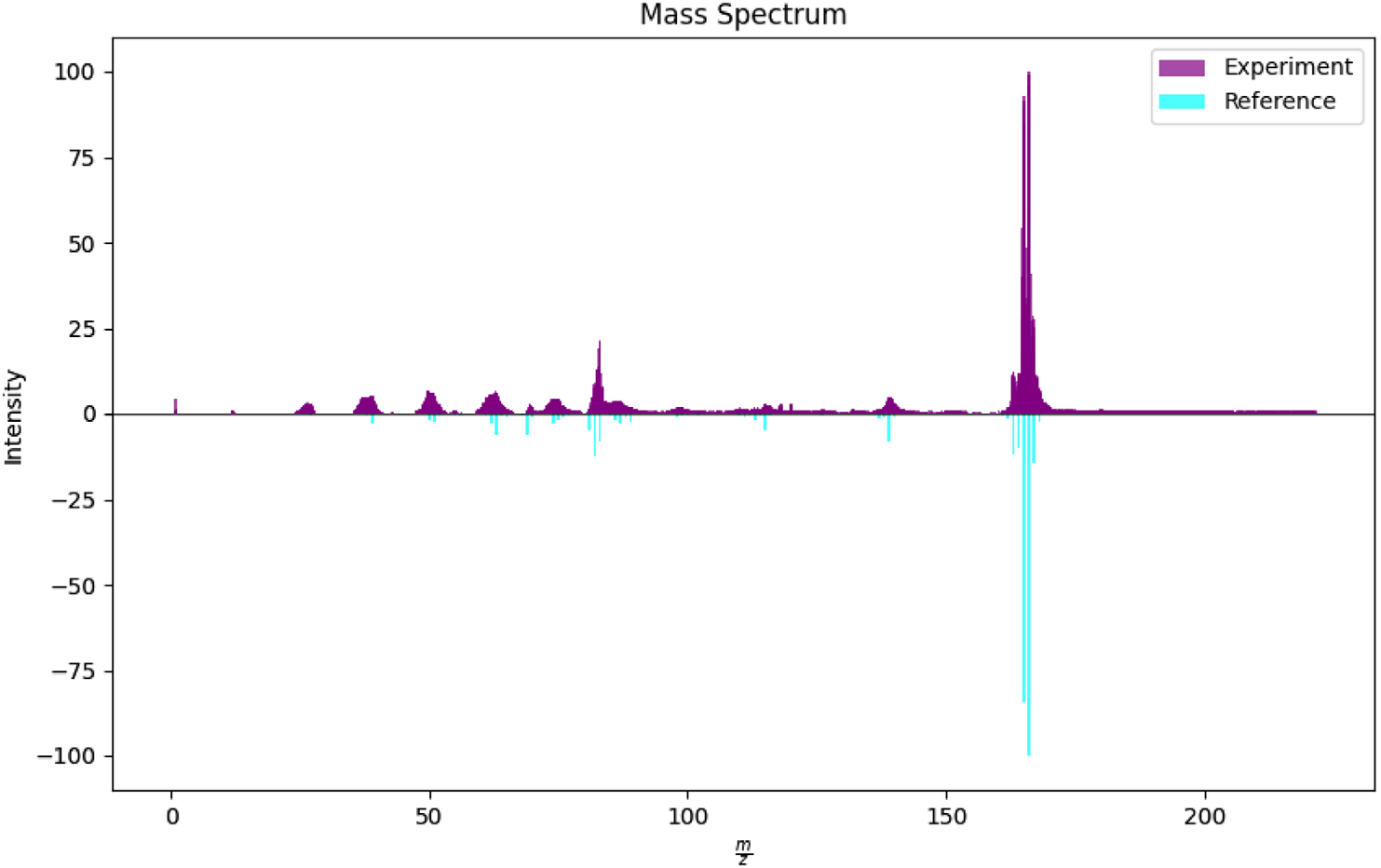
Comparison of the experimental and reference spectrum of fluorene for the highest ($6.8\times 10^{13}\;\text{W}/{\text{cm}}^{2}$) peak power mass spectra.

The background removal results are shown in Figure 8. As one can see, the background is indeed removed quite efficiently, leaving only the signals from the ion fragments. The cleaning in the range of higher masses is somewhat less effective, which is due to the overall background level increase, as clearly seen in a logarithmic plot. Nevertheless, such background removal was sufficient to identify fluorene in the case of all experimental spectra considered in this work. The results for the highest peak power spectrum are shown in Figure 9.

### 3.4 Performance Tests With an Experimental Dataset of Cleaned Spectra

The mass spectra of 64 substances were recorded using GC (HP6890, Agilent Technologies) coupled to a single quadrupolar MS (HP5972A or HP5973, Agilent Technologies) or with GC (Trace 1310) coupled to MS (TSQ Duo Triple Quadrupole, Thermo Scientific). Helium was used as a carrier gas, and the spectra were measured in the range of 50–500  m/z. The EI was used to ionize species with an electron KE of 70 eV. More details on the measurement parameters are available in ESI.

The experimental dataset consists of several classes of substances: Acid contaminants, chlorophenols, dioxins, PAHs, pesticides, and herbicides. For each of the compounds from this dataset, the reference spectrum was added to the database, and then ToxicMassSceptic was tested to provide the assignment results. We ranked the performance in each dataset using six scores: Top‐1, top‐3, top‐5, and top‐10 accuracies from Equation (17), MRR from Equation (18), and MR from Equation (19). The results of the test are given in Table 4. As one can see, most of the species were correctly identified in the top‐3 best‐matched substances, and the correct compound was the best‐matched one 60% of the time, on average. With that, we conclude that the current performance allows the identification of species in unknown samples.

**TABLE 4:** Performance of the ToxicMassSceptic assignment algorithm on the experimental datasets of various classes of substances.

Substance class	$N_{\text{subst}}$	Top‐1	Top‐3	Top‐5	Top‐10	MRR	MR
Acid contaminants	9	44.4	55.6	55.6	66.7	52.8	17.0
Dioxins	4	75.0	100.0	100.0	100.0	87.5	1.2
PAHs	16	43.8	100.0	100.0	100.0	68.8	1.8
Pesticides	29	82.8	100.0	100.0	100.0	90.8	1.2
Herbicides	6	50.0	83.3	83.3	83.3	66.8	19.2

### 3.5 Testing Theoretical Reference Against Cleaned Experimental Data

In the dataset used in Section 3.4, there were three dioxines: 1,2‐Dichlorodibenzo‐p‐dioxin, 1,3‐Dichlorodibenzo‐p‐dioxin, and 1,4‐Dichlorodibenzo‐p‐dioxin. These compounds are suitable for testing the assignment of experimental spectra against theoretically predicted mass spectra. For that reason, we computed the theoretical mass spectra of these three structural isomers using the workflow shown in Figure 2. In addition to that, in Section 3.1, we calculated theoretical mass spectra for o‐chlorophenoxyacetic acid and vinclozolin, which were also present in the same database.

Thus, we took these five substances to test their identification with the ToxicMassSceptic software. The resulting ranking of these theoretical spectra (R) against their experimental counterparts is given in Table 5 in columns Threshold=0%. As one can see, the results are acceptable. However, upon examination of the theoretical spectra, one can see that the number of reference lines ($N_{\text{lines}}$) is much larger than usually available for experimental spectra taken from various databases (which is typically of the order of a few tens of data points). Therefore, we have tried to remove some of the fragments with lower intensities from the theoretical spectra to see the effect on the identification of substances. In particular, we removed every lower‐intensity peak by setting a relative threshold with respect to the most intensive one. We tried two settings: Thresholds of 1% and 5%, which drastically reduced the number of lines and had an effect on the prediction performance (see Table 5). With a 1% threshold, the MR value for this set of five spectra was slightly lower than at the 0% and 5% settings, which indicates that there is an optimal number of lines to represent a species in the database, as too many or too few may lead to misidentification of the species. Therefore, we recommend removing the weak intensity fragments when using ToxicMassSceptic for predicting theoretical mass spectra, as this improves the identification probability P(B|A) (Equation 9). The importance of the latter can be seen from the definition of the metametric from Equation (15).

**TABLE 5:** Ranking (R) of and the number of lines ($N_{\text{lines}}$) in the theoretically predicted mass spectra of five substances against the experimental data. Different threshold values denote the removal of the weak intensity peaks from the reference dataset. 1,2‐DpD, 1,3‐DpD, 1,4‐DpD, and o‐CA denote 1,2‐Dichlorodibenzo‐p‐dioxin, 1,3‐Dichlorodibenzo‐p‐dioxin, 1,4‐Dichlorodibenzo‐p‐dioxin, and o‐chlorophenoxyacetic, respectively. The last row is the MR [see Equation (19)] values for the dataset of these five molecules at a given threshold.

Substance	Threshold=0%		Threshold=1%		Threshold=5%	
	R	$N_{\text{lines}}$	R	$N_{\text{lines}}$	R	$N_{\text{lines}}$
1,2‐DpD	21	154	13	64	11	15
1,3‐DpD	37	135	20	64	31	15
1,4‐DpD	10	152	3	69	11	17
o‐CA	5	140	3	45	3	19
Vinclozolin	2	201	14	26	36	7
MR	15.0		10.6		18.4	

## 4 Conclusions

In this article, we have presented an algorithm and a computer program for identifying toxic and combat compounds using mass spectrometry, ToxicMassSceptic, that is easy to operate for nonprofessionals. An essential part of it is the database of substances, assembled from multiple different sources, most prominently from databases like the NIST Chemistry WebBook and the SDBS of AIST, as well as from quantum chemical modeling. The use of theoretically predicted mass spectra allowed us to obtain reference data for poisonous substances for which no publicly accessible data exist. According to our tests against simulated and experimental datasets, ToxicMassSceptic with the database can facilitate preliminary identification of possible traces of poisonous and explosive substances. However, the current approach implies that the best matching result is always given. This is due to the open problem of finding thresholds for the current definition of the metametric. Therefore, the identification results are always biased toward the available database. The preliminary analysis results should always invoke a manual inspection for a few of the best‐matching substances, to check that the identified peaks are indeed present in the spectra. The final conclusions regarding substance identification should always be based on expert opinion and validated with other experimental methods, such as NMR or rotational spectroscopy [95].

## Author Contributions

Conceptualization, **Denis S. Tikhonov:** methodology, **Denis S. Tikhonov: Denis G. Artiukhin:** and **Vladimir V. Rybkin:** software, **Denis S. Tikhonov: Alexander A. Maryewski: Aleksandr A. Avdoshin: Olgert Dallakyan: Vladimir V. Rybkin: Denis G. Artiukhin:** validation, **Denis S. Tikhonov: Denis G. Artiukhin**: **Vladimir V. Rybkin:** formal analysis, **Denis S. Tikhonov: Alexander A. Maryewski: Vladimir V. Rybkin: Denis G. Artiukhin:** investigation, **Denis S. Tikhonov: Mikhail A. Kalinin: Alexander A. Maryewski: Aleksandr A. Avdoshin: Olgert Dallakyan: Nikita A. Vasilev: Egor A. Eliseev: Mandy Koch:** data curation, **Denis S. Tikhonov: Mikhail A. Kalinin:** **Aleksandr A. Avdoshin**: **Nikita A. Vasilev: Egor A. Eliseev: Mandy Koch: Vladimir V. Rybkin:** writing – original draft preparation, **Denis S. Tikhonov:** writing – review and editing, **Alexander A. Maryewski: Denis G. Artiukhin: Vladimir V. Rybkin:** visualization, **Denis S. Tikhonov: Aleksandr A. Avdoshin: Olgert Dallakyan:** supervision, **Denis S. Tikhonov: Denis G. Artiukhin: Vladimir V. Rybkin:** project administration, **Denis S. Tikhonov: Denis G. Artiukhin: Vladimir V. Rybkin:** All authors have read and agreed to the published version of the manuscript.

## Conflicts of Interest

The authors declare no conflicts of interest.

## Supporting information


**Data S1.** Supporting Information.

## Data Availability

The latest version of the software can be obtained from the GitLab repository https://gitlab.com/madschumacher/toxicmasssceptic/. A stable version of the software and database is also available in the ESI. Besides the software, the ESI information on the experimental conditions for GC‐MS measurements and the measurements themselves, procedures for manual digitizing of mass spectra from NIST Chemistry WebBook, the results of the statistical testing of ToxicMassSceptic on the generated dataset, and the simulated mass spectra. The full simulations of the mass spectra used here can be obtained from the Zenodo repository: https://dx.doi.org/10.5281/zenodo.14831652.
