# Supplementary material for: Simplistic Software for Analyzing Mass Spectra and a Mixed Experimental‐Theoretical Database for Identifying Poisonous and Explosive Substances
Source: J Comput Chem. 2025 Jun 25;46(17):e70148. doi: 10.1002/jcc.70148 (PMC12188633; doi:10.1002/jcc.70148)
Supplement: Supplementary file 1 — Data S1. Supporting Information. [file JCC-46-0-s001.zip › Digitizing_MassSpectra_from_NIST_Chemistry_WebBook.pdf]

Digitization of mass spectra from the NIST Chemistry WebBook database

Introduction

The excellent NIST Chemistry WebBook database has made its mass spectra database unavailable for download (due to commercialization). However, the spectra themselves are still available for viewing. So you can pull them out manually. 🙂 🙂

How to digitize these spectra?

Step 1: find this substance in the database. To do this, open the database (https://webbook.nist.gov/chemistry/):

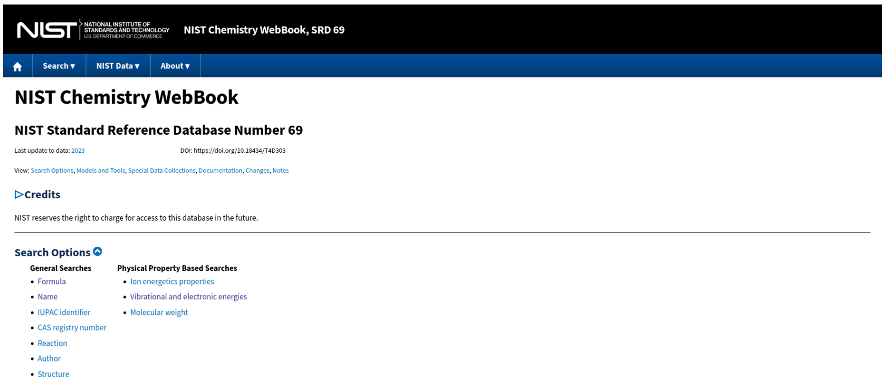

and click either on “Formula,” where we are asked to enter the empirical formula of the molecule in question (say, HCN):

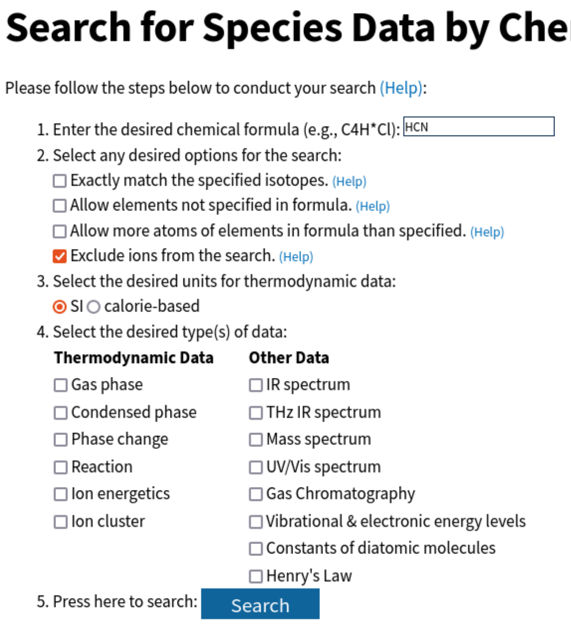

or on “Name,” in which case we can enter the name of the molecule (for HCN, it is “hydrogen cyanide”):

Search for Species Data by Chemical Name

Please follow the steps below to conduct your search (Help):

1. Enter a chemical species name or pattern: (e.g., methane, *2-hexene) Hydrogen cyanide
2. Select the desired units for thermodynamic data:
   ◉ SI ○ calorie-based
3. Select the desired type(s) of data:

Thermodynamic Data	Other Data
☐ Gas phase	☐ IR spectrum
☐ Condensed phase	☐ THz IR spectrum
☐ Phase change	☐ Mass spectrum
☐ Reaction	☐ UV/Vis spectrum
☐ Ion energetics	☐ Gas Chromatography
☐ Ion cluster	☐ Vibrational & electronic energy levels
	☐ Constants of diatomic molecules
	☐ Henry's Law

4. Press here to search: Search

Once a compound has been found, open the “Mass spectrum” section (in “Other data available”):

Hydrogen cyanide

- **Formula:** CHN
- **Molecular weight:** 27.0253
- **IUPAC Standard InChI:** InChI=1S/CHN/c1-2/h1H InChI v 1.06
- **IUPAC Standard InChIKey:** LELOWRISYMNNSU-UHFFFAOYSA-N
- **CAS Registry Number:** 74-90-8
- **Chemical structure:**

  This structure is also available as a 2d Mol file
- **Other names:** Hydrocyanic acid; AC; Blausaeure (German); Carbon hydride nitride (CHN); Formic anammoni Cyanwasserstoff; Cyclone B; Cyjanowodor; Evercyn; NA 1051; Prussic acid, unstabilized; Rcra waste number F
- **Permanent link** for this species. Use this link for bookmarking this species for future reference.
- **Information on this page:**
  - Notes
- **Other data available:**
  - Gas phase thermochemistry data
  - Condensed phase thermochemistry data
  - Phase change data
  - Reaction thermochemistry data: reactions 1 to 50, reactions 51 to 92
  - Henry's Law data
  - Gas phase ion energetics data
  - Ion clustering data
  - IR Spectrum
  - Mass spectrum (electron ionization)
  - Vibrational and/or electronic energy levels
  - Gas Chromatography

Step 2: Manually pull the spectrum found. To do this, we look at the spectrum view where it is given to us. On the *x*-axis is the mass-per-charge (*m*/*z*), which is a dimensionless quantity according to IUPAC. On the *y*-axis is the intensity, where the intensity of the maximum peak is taken as 100:

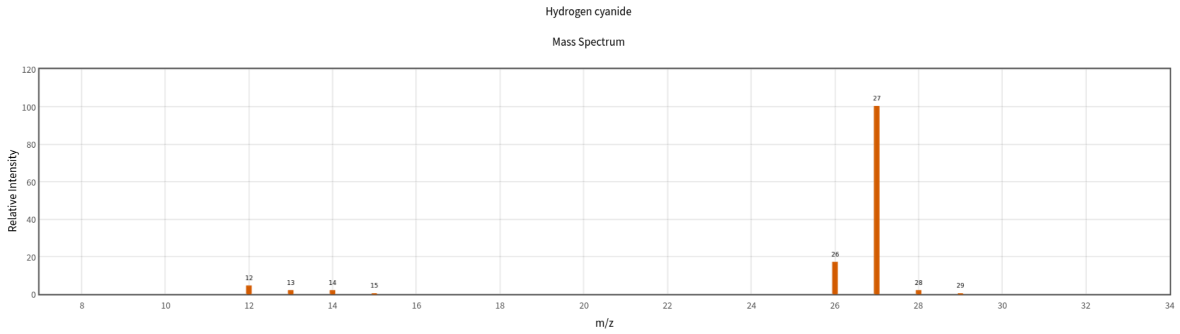

Above each peak is signed its *m*/*z* value, so all you have to pull out is the peak intensity. All peaks with intensity greater than 1 (i.e., 1% of the maximum peak) should be included for the minimum case. The rest can be ignored, but if you want to be a hero, you're welcome to finish all of them.

To get the intensity, you can zoom in on the part of the spectrum of interest by selecting the desired area with the left mouse button. Left-clicking without selection restores the original full scale.

To record in the minimum version, you have to be accurate up to the whole part of the number. As a result of such digitization, we will get two columns of numbers of the following kind (example - HCN):

m/z	intensity
28	2
27	100
26	17
14	2
13	2
12	4

If we compare this to what we were able to download before NIST removed the download, we see:

m/z	intensity
12	4.2
13	1.7
14	1.7
15	0.1
26	16.8
27	100
28	1.7
29	0.1

I.e., quite a good match. The resulting tables can either be saved in Excel tables or as a text file. In the database itself, which already exists, you need to create a folder with the empirical formula, for example, according to Hill's system (first carbon, then hydrogen, then all other elements alphabetically), and then some molecule identifier through the bottom dash. Inside should be a ref.ms file, which is a two-column file.

Оцифровка масс-спектров с базы данных NIST Chemistry WebBook

Введение

Замечательная база данных [NIST Chemistry WebBook](https://webbook.nist.gov/chemistry/) сделала свою базу данных масс-спектров недоступной для скачивания (в связи с её коммерциализацией). Тем не менее, сами спектры всё ещё доступны для просмотра. Поэтому их можно вытащить вручную. 🙂

Как эти спектры оцифровать?

Шаг 1: найти это соединение в базе данных. Для этого открываем базу (https://webbook.nist.gov/chemistry/):

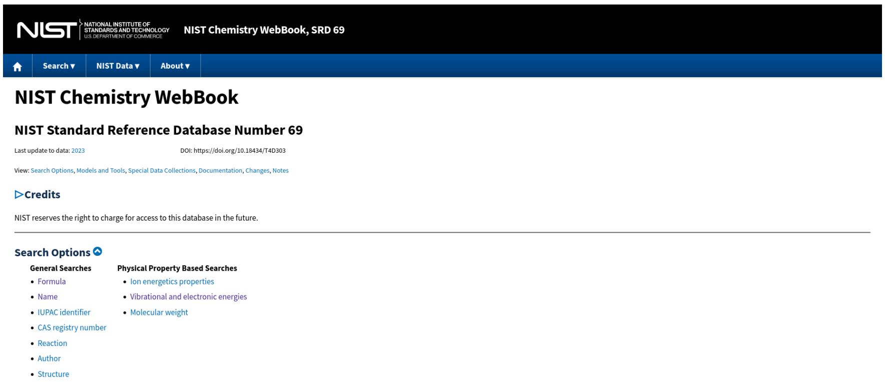

и нажимаем или на “Formula,” где от нас потребуется ввести брутто-формулу соответствующей молекулы (скажем, HCN):

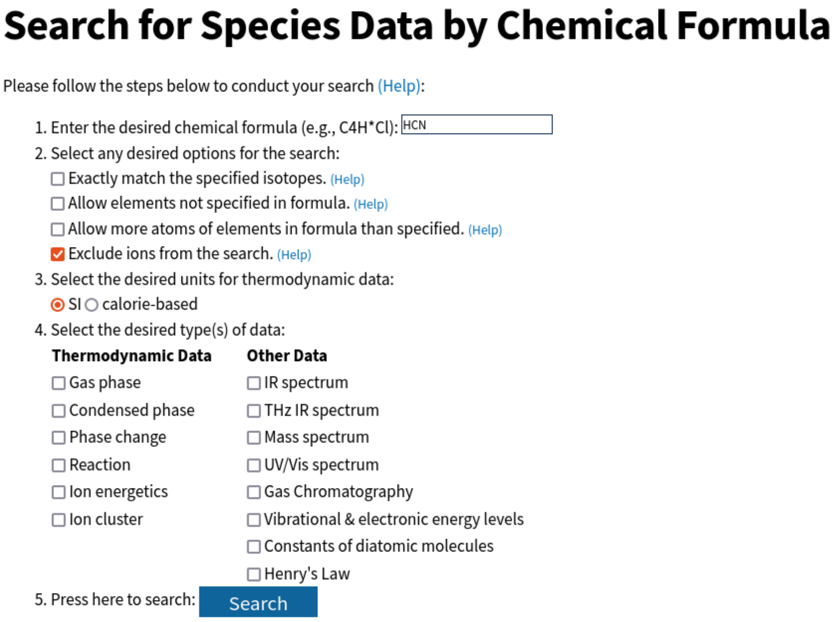

или же на “Name,” и в этом случае можно ввести название молекулы (для HCN это “hydrogen cyanide”:

Search for Species Data by Chemical Name

Please follow the steps below to conduct your search (Help):

1. Enter a chemical species name or pattern: (e.g., methane, *2-hexene) [Hydrogen cyanide]
2. Select the desired units for thermodynamic data:
   ◉ SI ○ calorie-based
3. Select the desired type(s) of data:

**Thermodynamic Data**	**Other Data**
☐ Gas phase	☐ IR spectrum
☐ Condensed phase	☐ THz IR spectrum
☐ Phase change	☐ Mass spectrum
☐ Reaction	☐ UV/Vis spectrum
☐ Ion energetics	☐ Gas Chromatography
☐ Ion cluster	☐ Vibrational & electronic energy levels
	☐ Constants of diatomic molecules
	☐ Henry's Law

4. Press here to search: [Search]

После того, как нашлось соединение, открываем раздел “Mass spectrum” (в “Other data available”):

Hydrogen cyanide

- **Formula:** CHN
- **Molecular weight:** 27.0253
- **IUPAC Standard InChI:** InChI=1S/CHN/c1-2/h1H InChI v 1.06
- **IUPAC Standard InChIKey:** LELOWRISYMNNSU-UHFFFAOYSA-N
- **CAS Registry Number:** 74-90-8
- **Chemical structure:**
  This structure is also available as a 2d Mol file
- **Other names:** Hydrocyanic acid; AC; Blausaeure (German); Carbon hydride nitride (CHN); Formic anammoni Cyanwasserstoff; Cyclone B; Cyjanowodor; Evercyn; NA 1051; Prussic acid, unstabilized; Rcra waste number F
- **Permanent link** for this species. Use this link for bookmarking this species for future reference.
- **Information on this page:**
  - Notes
- **Other data available:**
  - Gas phase thermochemistry data
  - Condensed phase thermochemistry data
  - Phase change data
  - Reaction thermochemistry data: reactions 1 to 50, reactions 51 to 92
  - Henry's Law data
  - Gas phase ion energetics data
  - Ion clustering data
  - IR Spectrum
  - Mass spectrum (electron ionization)
  - Vibrational and/or electronic energy levels
  - Gas Chromatography

Шаг 2: вручную вытащить найденный спектр. Для этого мы смотрим на просмотр спектра, где он нам дан. По оси *x* находится масса-на-заряд (*m*/*z*), что по ИЮПАК является безразмерной величиной. По оси *y* находится интенсивность, где интенсивность максимального пика принята за 100:

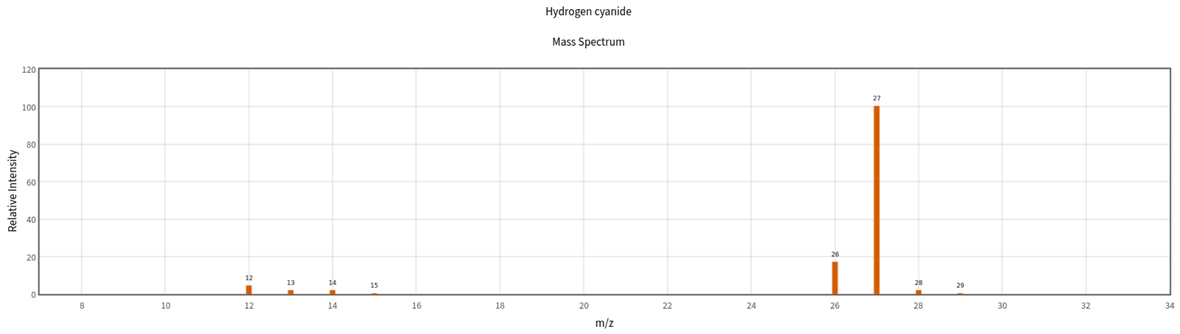

Над каждым пиком подписано его значение *m/z*, поэтому всё, что нужно вытащить – это интенсивность пика. Для минимального случая надо внести все пики с интенсивностью больше 1 (т.е. 1% от максимального пика). Остальное можно проигнорить, но если хочется погеройствовать – пожалуйста.

Чтобы получить интенсивность, можно увеличить интересующую часть спектра, выделяя нужную область левой клавишей мышки. Нажатие левой клавишей без выделения восстанавливает изначальный полный масштаб.

Записывать в минимальном варианте нужно с точностью до целых. В результате такой оцифровки мы получим две колонки чисел следующего вида (пример – HCN):

m/z	интенсивность
28	2
27	100
26	17
14	2
13	2
12	4

Если сравнить это с тем, что удалось скачать до убирания скачки NIST-ом, то увидим:

m/z	интенсивность
12	4.2
13	1.7

14	1.7
15	0.1
26	16.8
27	100
28	1.7
29	0.1

Т.е. вполне неплохое совпадение. Полученные таблицы можно или сохранять в Экселевских таблицах, или в виде текстового файла. В самой базе данных, которая уже есть, нужно создать папку с брутто-формулой, например, по системе Хилла (сначала углерод, потом водороды, потом все остальные элементы по алфавиту), и потом некий идентификатор молекулы через нижний прочерк. Внутри же должен быть файлик ref.ms, который из себя представляет соответствующий двухколоночный файл.
