# Supplementary material for: Simplistic Software for Analyzing Mass Spectra and a Mixed Experimental‐Theoretical Database for Identifying Poisonous and Explosive Substances
Source: J Comput Chem. 2025 Jun 25;46(17):e70148. doi: 10.1002/jcc.70148 (PMC12188633; doi:10.1002/jcc.70148)
Supplement: Supplementary file 1 — Data S1. Supporting Information. [file JCC-46-0-s001.zip › GC-MS_measurement_parameters.pdf]

- all analytes were measured on GC-MS or GC-MS/MS (tables with parameters named like sheets in „Massenspektren Kalinin.xlsx“)

For: „Dioxine“

Parameter	Conditions/Dimensions
**Instrument**	GC (HP 6890, Agilent Technologies) coupled with mass spectrometer (HP5972A, Agilent Technologies) single Quad
**Column**	SLB-5ms: 30 m x 0.25 mm i.d., 0.25 µm film thickness (Supelco)
**Carrier Gas**	helium, 1 mL/min constant flow
**Injection Temperature**	250 °C
**Injection Mode**	splitless,<br>split@1 min = 61.2 mL/min,<br>gas saver @4 min = 15 mL/min
**Injection Volume**	1 µL
**Oven program**	50 °C (hold 1 min)<br>with 10 K/min to 150 °C (hold 5 min)<br>with 3 K /min to 260 °C (hold 10 min)<br>with 25 K / min to 280 °C (hold 5 min)
**Transferline Temperature**	250 °C
**MS Source Temperature**	230 °C
**MS Quad Temperature**	150 °C
**Aquisition Mode**	EI Fullscan
**Ionisation energy**	70 eV
**Solvent Delay**	10 min
**Mass range**	50 – 500 m/z

For: „Pesticides I“

Parameter	Conditions/Dimensions
**Instrument**	GC (HP 6890, Agilent Technologies) coupled with mass spectrometer (HP5973, Agilent Technologies) single Quad
**Column**	VF-5ms: 30 m x 0.25 mm i.d., 0.25 µm film thickness with 5 m EZ guard column (Agilent J&W)
**Carrier Gas**	helium, 1 mL/min constant flow
**Injection Temperature**	270 °C
**Injection Mode**	splitless,<br>split@0.5 min = 50 mL/min,<br>gas saver @4 min = 15 mL/min
**Injection Volume**	1 µL
**Oven program**	55 °C (hold 3 min)<br>with 40 K/min to 120 °C<br>with 4 K /min to 250 °C (hold 20 min)<br>with 45 K / min to 280 °C (hold 5 min)
**Transferline Temperature**	270 °C
**MS Source Temperature**	230 °C
**MS Quad Temperature**	150 °C
**Aquisition Mode**	EI Fullscan
**Ionisation energy**	70 eV
**Solvent Delay**	20 min
**Mass range**	50 – 550 m/z

For: „Pesticides II“

Parameter	Conditions/Dimensions
**Instrument**	GC (HP 6890, Agilent Technologies) coupled with mass spectrometer (HP5973, Agilent Technologies) single Quad
**Column**	VF-5ms: 30 m x 0.25 mm i.d., 0.25 µm film thickness (Agilent J&W)
**Carrier Gas**	helium, 1 mL/min constant flow
**Injection Temperature**	200 °C
**Injection Mode**	splitless,<br>split@1 min = 50 mL/min,<br>gas saver @4 min = 15 mL/min
**Injection Volume**	1 µL
**Oven program**	60 °C (hold 2 min)<br>with 20 K/min to 120 °C<br>with 4 K /min to 280 °C (hold 10 min)
**Transferline Temperature**	250 °C
**MS Source Temperature**	230 °C
**MS Quad Temperature**	150 °C
**Aquisition Mode**	EI Fullscan
**Ionisation energy**	70 eV
**Solvent Delay**	20 min
**Mass range**	50 – 550 m/z

For: „PAHs“

Parameter	Conditions/Dimensions
**Instrument**	GC (HP 6890, Agilent Technologies) coupled with mass spectrometer (HP5972A, Agilent Technologies) single Quad
**Column**	SLB-5ms: 30 m x 0.25 mm i.d., 0.25 µm film thickness (Supelco)
**Carrier Gas**	helium, 1 mL/min constant flow
**Injection Temperature**	250 °C
**Injection Mode**	splitless,<br>split@ 1 min = 50 mL/min,<br>gas saver @4 min = 15 mL/min
**Injection Volume**	1 µL
**Oven program**	95 °C (hold 1 min)<br>with 7 K /min to 300 °C (hold 15 min)
**Transferline Temperature**	280 °C
**MS Source Temperature**	230 °C
**MS Quad Temperature**	150 °C
**Aquisition Mode**	EI Fullscan
**Ionisation energy**	70 eV
**Solvent Delay**	5 min
**Mass range**	50 – 520 m/z

For „acidic contaminants“ (derivatized to PFB ester (m/z of PFB-group: 181))

**Parameter**	**Conditions/Dimensions**
**Instrument**	GC (Trace 1310, ThermoScientific) coupled with mass spectrometer (TSQ Duo, ThermoScientific) TripleQuad
**Column**	VF-5ms: 30 m x 0.25 mm i.d., 0.25 µm film thickness with 5 m EZ guard column (Agilent J&W)
**Carrier Gas**	helium, 1 mL/min constant flow
**Injection Temperature**	280 °C
**Injection Mode**	splitless,<br>split@ 2 min = 50 mL/min,<br>gas saver @4 min = 15 mL/min
**Injection Volume**	1 µL
**Oven program**	95 °C (hold 1 min)<br>with 30 K/min to 170 °C<br>with 2 K/min to 190 °C (hold 5 min)<br>with 7 K /min to 270 °C<br>with 30 K /min to 310 °C (hold 3 min)
**Transferline Temperature**	280 °C
**MS Source Temperature**	300 °C
**MS Quad Temperature**	not specified
**Aquisition Mode**	EI Fullscan
**Ionisation energy**	70 eV
**Solvent Delay**	15 min
**Mass range**	50 – 550 m/z

For „Phenoxy herbicides“ (derivatized to PFB ester (m/z of PFB-group: 181))

Parameter	Conditions/Dimensions
**Instrument**	GC (HP 6890, Agilent Technologies) coupled with mass spectrometer (HP5972A, Agilent Technologies) single Quad
**Column**	VF-5ms: 30 m x 0.25 mm i.d., 0.25 µm film thickness with 5 m EZ guard column (Agilent J&W)
**Carrier Gas**	helium, 0.8 mL/min constant flow
**Injection Temperature**	250 °C
**Injection Mode**	splitless split@ 1 min = 61.2 mL/min, gas saver @2 min = 15 mL/min
**Injection Volume**	1 µL
**Oven program**	95 °C (hold 1 min)<br>with 40 K/min to 170 °C<br>with 3 K/min to 205 °C<br>with 25 K /min to 250 °C<br>with 20 K /min to 275 °C (hold 10 min)
**Transferline Temperature**	270 °C
**MS Source Temperature**	200 °C
**MS Quad Temperature**	150 °C
**Aquisition Mode**	EI Fullscan
**Ionisation energy**	70 eV
**Solvent Delay**	7 min
**Mass range**	50 – 550 m/z

For „chlorophenols" (derivatized with trimethylsilylether to TMS derivatives)

Parameter	Conditions/Dimensions
**Instrument**	GC (HP 6890, Agilent Technologies) coupled with mass spectrometer (HP5972A, Agilent Technologies) single Quad
**Column**	VF-5ms: 30 m x 0.25 mm i.d., 0.25 µm film thickness with 5 m EZ guard column (Agilent J&W)
**Carrier Gas**	helium, 0.8 mL/min constant flow
**Injection Temperature**	250 °C
**Injection Mode**	splitless split@ 1 min = 61.2 mL/min, gas saver @2 min = 15 mL/min
**Injection Volume**	1 µL
**Oven program**	60 °C (hold 2 min)<br>with 10 K/min to 260 °C (hold 10 min)
**Transferline Temperature**	270 °C
**MS Source Temperature**	200 °C
**MS Quad Temperature**	150 °C
**Aquisition Mode**	EI Fullscan
**Ionisation energy**	70 eV
**Solvent Delay**	6.5 min
**Mass range**	50 – 550 m/z
