# Supplementary material for: Simplistic Software for Analyzing Mass Spectra and a Mixed Experimental‐Theoretical Database for Identifying Poisonous and Explosive Substances
Source: J Comput Chem. 2025 Jun 25;46(17):e70148. doi: 10.1002/jcc.70148 (PMC12188633; doi:10.1002/jcc.70148)

Methanol
(CH_3OH)

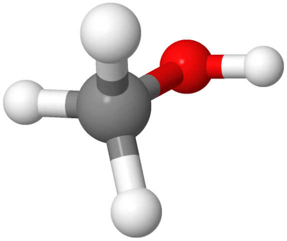

Novichok A-230
($C_7H_{16}FN_2OP$)

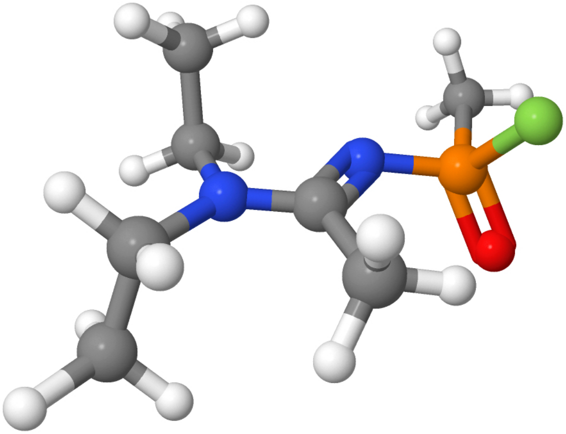

o-Chlorophenoxyacetic acid
($C_8H_7ClO_3$)

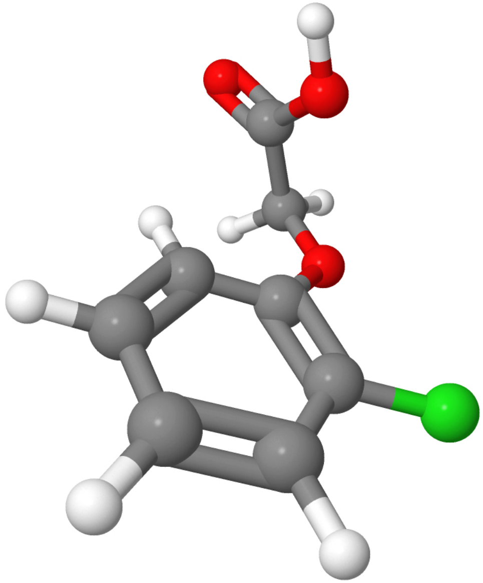

Vinclozolin
($C_{12}H_9Cl_2NO_3$)

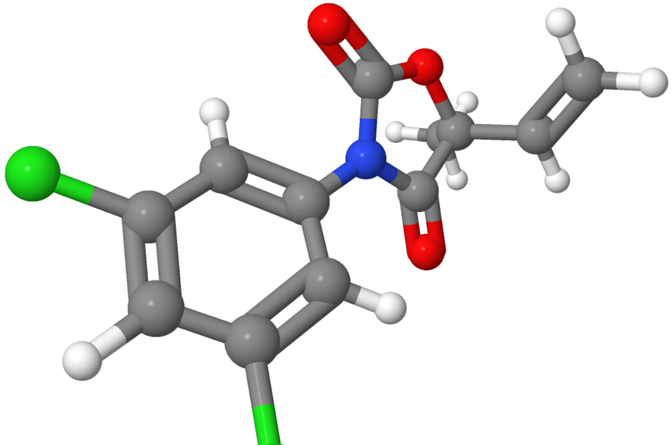

Supplement: Supplementary file 1 — Data S1. Supporting Information. [file JCC-46-0-s001.zip › abomd_simulation_results.zip!/abomd_simulation_results/plot_molecules/four_test_molecules.pdf]
