# Supplementary material for: Simplistic Software for Analyzing Mass Spectra and a Mixed Experimental‐Theoretical Database for Identifying Poisonous and Explosive Substances
Source: J Comput Chem. 2025 Jun 25;46(17):e70148. doi: 10.1002/jcc.70148 (PMC12188633; doi:10.1002/jcc.70148)
Supplement: Supplementary file 1 — Data S1. Supporting Information. [file JCC-46-0-s001.zip › abomd_simulation_results.zip!/abomd_simulation_results/C8H7ClO3_o-chlorophenoxyacetic_acid/step4_combine/o-chlorophenoxyacetic_acidcomparison_exp_vs_theory.pdf]

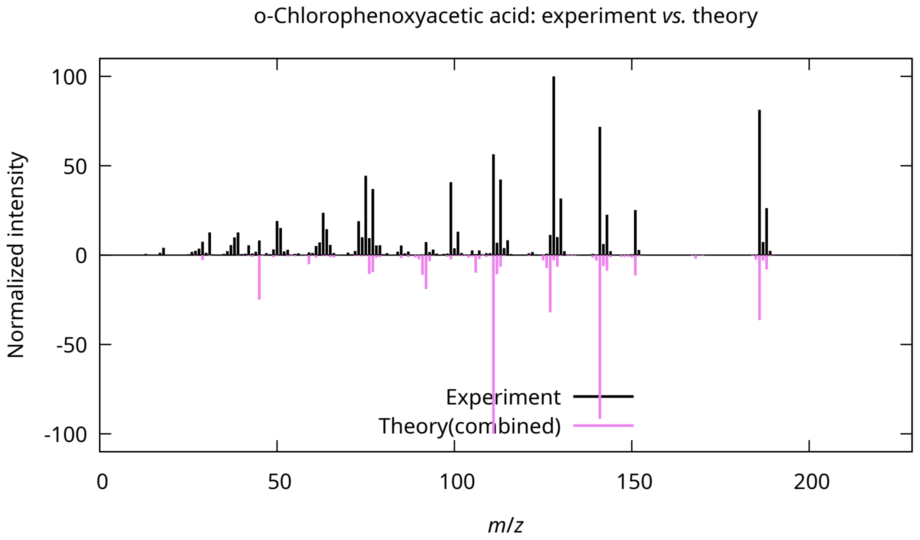
o-Chlorophenoxyacetic acid: experiment vs. theory
100
50
0
-50
-100
Normalized intensity
0
50
100
150
200
m/z
Experiment
Theory(combined)
