# Supplementary material for: Simplistic Software for Analyzing Mass Spectra and a Mixed Experimental‐Theoretical Database for Identifying Poisonous and Explosive Substances
Source: J Comput Chem. 2025 Jun 25;46(17):e70148. doi: 10.1002/jcc.70148 (PMC12188633; doi:10.1002/jcc.70148)
Supplement: Supplementary file 1 — Data S1. Supporting Information. [file JCC-46-0-s001.zip › abomd_simulation_results.zip!/abomd_simulation_results/CH3OH_methanol/step4_combine/methanol_comparison_qcxms_vs_dissmd.pdf]

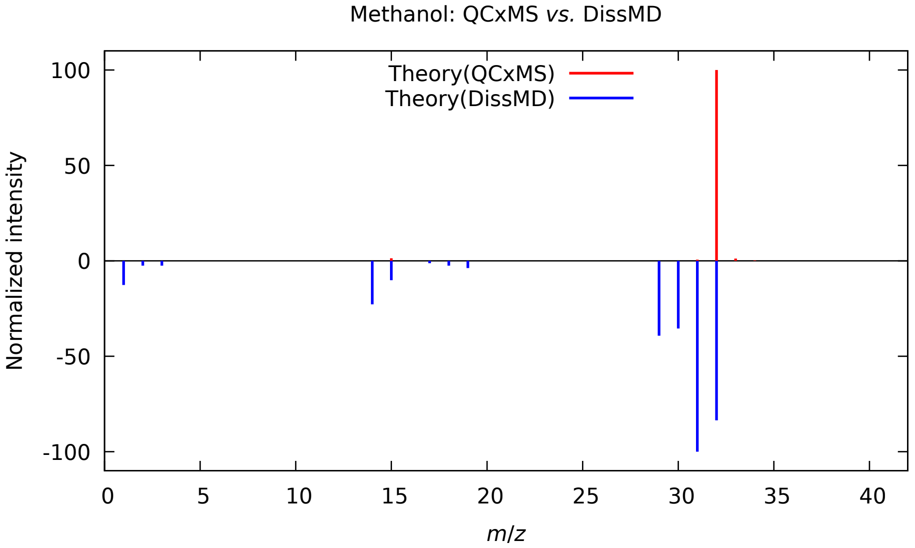
Methanol: QCxMS *vs.* DissMD
Theory(QCxMS)
Theory(DissMD)
Normalized intensity
100
50
0
-50
-100
0
5
10
15
20
25
30
35
40
m/z
